# The Right Stuff: Getting the right data at the right time and using that data to drive evidence‐based practice and policy

**DOI:** 10.1002/lrh2.10432

**Published:** 2024-05-27

**Authors:** Lucy A. Savitz, Sarah M. Greene, Michael K. Gould, Harold S. Luft

**Affiliations:** ^1^ Department of Health Policy and Management University of Pittsburgh School of Public Health Pittsburgh Pennsylvania USA; ^2^ UPMC Health Plan's Center for High Value Health Care Pittsburgh Pennsylvania USA; ^3^ National Academy of Medicine Washington DC USA; ^4^ Department of Health Systems Science Kaiser Permanente Bernard J. Tyson School of Medicine Pasadena California USA; ^5^ Caldwell B. Esselstyn Professor Emeritus of Health Policy and Health Economics, Philip R. Lee Institute for Health Policy Studies, University of California, San Francisco San Francisco California USA

When researchers are embedded within healthcare systems and collaborate with practitioners and operational leaders, they may be able to rapidly identify problems and opportunities that can be addressed to improve quality and affordability. While other industries have well‐developed data exploration processes (e.g., banking), healthcare is still developing its methods with widely varying data sources, huge quantities of unstructured data, uncertain precision in measurement, uncertainties about data quality, and complicated and stringent regulations and policies on data access. In recognition of these challenges, the AcademyHealth Learning Health System (LHS) Interest Group (In 2021, *Learning Health Systems* journal established a formal relationship with AcademyHealth, serving as the official journal of its LHS Interest Group.) released a call for papers in June 2023 to focus on challenges encountered by investigators related to the use of real‐world data in embedded research.

We use the term “embedded researcher” to characterize a broad range of people well‐trained in research methods using real‐world data. Being located inside a health system, they often have privileged access to data and the practitioners who may be observing new situations, problems, or opportunities for improvement. Unlike colleagues only involved in internal quality improvement efforts, embedded researchers also seek to broadly share their findings and create generalizable knowledge. The sharing is less focused on the specific findings—too many things may be unique about the setting, people, and other factors to be directly generalizable. The challenges faced and techniques used to overcome them, however, may offer important lessons for other embedded researchers.

As LHSs mature and internally tackle increasingly complex problems with embedded research, the challenges presented in using real‐world data for locally applied health services research are important to understand. Taken together, the papers in this Special Issue offer insights into the frontiers of embedded research as LHSs embark on their own learning journey. Accelerating the transformation of data to knowledge requires an understanding of the underlying data and techniques needed to draw useful lessons from the data. Sharing experiences across teams and settings will help others in anticipating and addressing the challenges they are likely to encounter.

Toward that end, we invited papers for this thematic issue from the membership of AcademyHealth, the professional society for health services research. The membership is truly transdisciplinary and represents a wide range of professionals working in a variety of settings. Thus, the compilation of articles in this Special Issue draws on diverse settings that describe multiple challenges around the novel data sources, delivery system structure, collaboration, and data infrastructure experienced by embedded researchers. After considerable review, three themes emerged:

Theme 1—application and integration of novel data sources: The experience report by Kogan and colleagues together with the commentary by Guo et al. and technical report by Clark et al. provide a broad view of the challenges encountered and progress made in drawing on the needed data and technical infrastructure to accomplish this work.

Theme 2—team dynamics and relationship to data and/or embedded research: Two experience reports by Branda et al. and Kennedy et al. are complemented by the Garcia et al. commentary and Wolf et al. technical report in illustrating the sociotechnical context issues that support embedded research.

Theme 3—dynamism of health care delivery and how it can affect data and/or research: The commentary by Savitz et al. together with the Larson et al. brief report and an illustrative research report from Progovac and colleagues underscore the role of operational needs in the design and methods used in embedded research using real world data.

As this Supplement illustrates, getting the right data at the right time (with the right interpretation) is imperative in producing the evidence needed for policy, practice, and research. These issues are illustrated via the contributed examples from the field. For all LHS efforts to reach their full potential, we need to develop the processes, standards, and methods necessary to fulfill the virtuous cycle of using evidence from embedded health services research for transformative improvements to healthcare and health.

